# Differential global and extra-cellular matrix focused gene expression patterns between normal and glaucomatous human lamina cribrosa cells

**Published:** 2009-01-15

**Authors:** Ruaidhrí P. Kirwan, Robert J. Wordinger, Abbot F. Clark, Colm J. O'Brien

**Affiliations:** 1 Conway Institute of Biomolecular and Biomedical Research, University College Dublin, Ireland; 2 Department of Cell Biology and Genetics, University of North Texas Health Center at Fort Worth, Fort Worth, TX; 3 Glaucoma Research, Alcon Research Ltd., Fort Worth, TX; 4 Department of Ophthalmic Surgery, Mater Misericordiae University Hospital, Dublin, Ireland

## Abstract

**Purpose:**

Marked extracellular matrix (ECM) remodeling occurs in the human optic nerve head in primary open angle glaucoma (POAG). The glial fibrillary acid protein (GFAP) negative lamina cribrosa cell may play an important role in this remodeling process. We report the first study of global and ECM-focused gene transcription differentials between GFAP-negative lamina cribrosa (LC) cells from normal and POAG human donors.

**Methods:**

GFAP-negative LC cell lines were generated from the optic nerve tissue of four normal (n=4) and four POAG (n=4) human donors. Using Affymetrix U133A arrays the transcriptional profile between the normal and diseased groups were compared. Bioinformatic analysis was performed using robust multichip average (RMA Express) and EASE/David. Real time TaqMan PCR and immunohistochemistry analyses were performed to validate the microarray data.

**Results:**

183 genes were upregulated by greater than 1.5 fold and 220 were down regulated by greater than 1.5 fold in the POAG LC cells versus normal controls. Upregulated genes in POAG LC cells included, transforming growth factor beta 1 (*TGFβ1*), secreted acid protein cysteine rich (*SPARC*), periostin (*POSTN*), thrombospondin-1 (*THBS1*), cartilage linking protein-1 (*CRTL-1*), and collagen type I  (*COL1A1*), collagen type V (*COL5A1*), and collagen type XI (*COL11A1*). Downregulated ECM genes in POAG included fibulin 1 (*FBLN1*), decorin (*DCN*), and collagen type XVIII (*COL18A1*). All TaqMan PCR validation assays were significant (*p<0.05) and consistent with the array data. Immunohistochemistry of one target (periostin) confirmed its differential expression at the protein level in POAG optic nerve head tissue compared with non-glaucomatous controls. Functional annotation and over-representation analysis identified ECM genes as a statistically over-represented class of genes in POAG LC cells compared with normal LC cells.

**Conclusions:**

This study reports for the first time that POAG LC cells in-vitro demonstrate upregulated ECM and pro-fibrotic gene expression compared with normal LC cells. This may be a pathological characteristic of this cell type in POAG in-vivo. We believe that the LC cell may be a pivotal regulator of optic nerve head ECM remodeling in POAG and an attractive target for molecular therapeutic strategies in the future.

## Introduction

Primary open angle glaucoma (POAG) is a sight threatening progressive optic neuropathy affecting 60 million people world wide [1]. Raised intraocular pressure (IOP) and reduced optic nerve head vascular perfusion are proposed risk factors for the development of this disorder [2,3]. One of the key pathological characteristics of POAG is fibrotic extracellular matrix (ECM) remodeling of the optic nerve head [4]. These ultrastructural changes include increased deposition of the proteins collagen I, IV and VI in addition to the synthesis of dysfunctional forms of elastin fiber [5]. This disturbed ECM metabolism is particularly obvious in the lamina cribrosa layer, where it is suspected of undermining the overall structural integrity of the optic nerve head (ONH) [6]. Mathematical models predict that chronically raised intraocular pressure above 30 mmHg will markedly compress the lamina cribrosa which may account for the characteristic 'cupped' morphology of the glaucomatous ONH [7]. Similarly, in-vivo primate models of POAG confirm this compression of the lamina cribrosa with consequent arrest of axoplasmic flow within its constituent retinal ganglion cell axons [8].

While the existence of ECM remodeling in the glaucomatous ONH is well documented, other aspects of the relevant molecular mechanism are still under study. A major area of interest centers on identifying the cell (or cells) responsible for producing this aberrant lamina cribrosa ECM [9]. One member of the glial cell population of the ONH that is emerging as a likely candidate is the GFAP-negative lamina cribrosa (LC) cell [10]. The LC cell is of relevance here because it bears similarities to myofibroblastic cells known to be responsible for fibrotic disease development elsewhere in the human body [11]. These similarities include constitutive expression of alpha-SMA, elastin, collagen type I, and fibronectin [12]. In addition, we have previously shown that the LC cell's capacity for TGF-β driven release of major modulators of fibrosis such as connective tissue growth factor (*CTGF*) and platelet derived growth factor-alpha (*PDFG-alpha*), points to a potentially pivotal role for this cell type in generalized wound healing in the ONH [13].

Other lines of enquiry have focused on the triggers for cellular release of fibrotic tissue and remodelling enzymes in the lamina cribrosa in POAG. In addition to molecular stimuli such as TGF- β, important roles for mechanical strain and hypoxia have been proposed in this context [14,15]. However, significant interplay between these diverse stimuli is a likely imperative in dictating the final glaucomatous phenotype. In probing the global gene expression changes in POAG LC cells we intended to search for genes concerned with fibrotic ECM changes but also others relevant to a more global pathological mechanism of POAG where neurodegeneration, hypoxic stress and cellular mechanical resilience may play a role. In this study, microarray analysis was used to define for the first time, a baseline global and ECM gene expression differential between POAG and normal LC cells. In so doing, a panel of established and potentially novel modulators of glaucomatous optic nerve head molecular pathology were identified.

## Methods

### In-vitro lamina cribrosa (LC) cell culture

**Figure 1:**
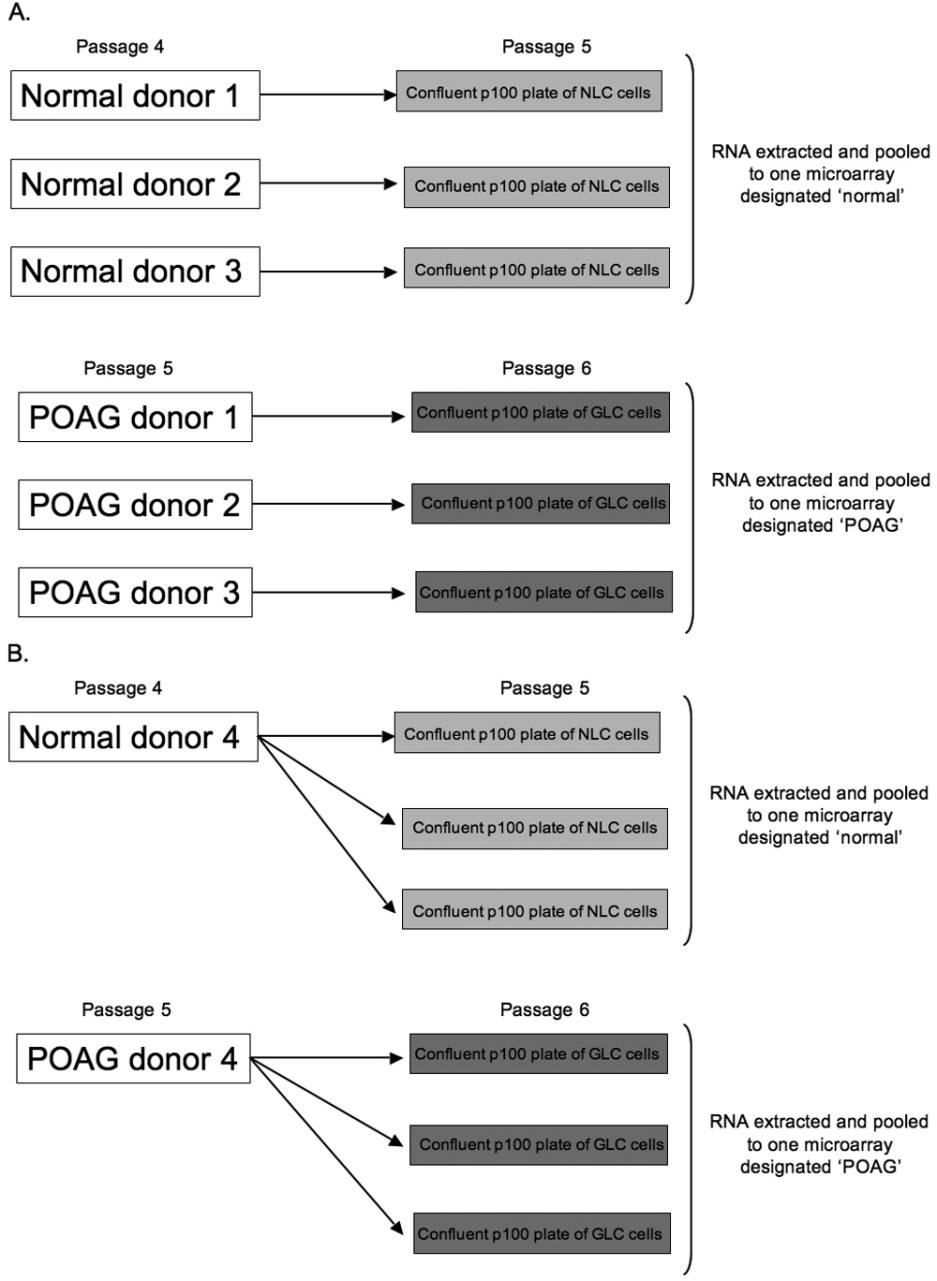
Microarray assay experimental design. For microarray analysis 1 (**A**), RNA from three separate normal (n=3) and three separate POAG (n=3) donor LC cell lines were pooled to individual 'normal' and 'POAG' microarrays respectively. In microarray analysis 1 LC cells from the normal and POAG donors were passaged in a 1:1 ratio. For microarray analysis 2 (**B**) a fourth normal and a fourth POAG donor provided RNA which was pooled to a second ‘normal’ and a second ‘POAG’ microarray respectively. In microarray analysis 2 LC cells from the normal and POAG donors were passaged in a 1:3 ratio. All four arrays were then normalized together and compared using Robust Multichip Average (RMA) software. The LC cells used were at passages 5 and 6. NLC=normal lamina cribrosa, GLC=POAG lamina cribrosa.

Twelve primary GFAP-negative lamina cribrosa (LC) cell lines were generated from human optic nerve head explants as described previously and supplied by Alcon Research Labs at Fort Worth, TX [12]. Six normal LC cell lines were from four donors with no history of eye disease and six POAG LC cell lines were from four donors with a documented history of POAG (Figure 1A,B). Cultures were maintained in DMEM (Sigma, Dorset, UK) supplemented with 10% fetal calf serum, penicillin (100 U/ml) and streptomycin (100 μg/ml) in a humidified incubator containing 5% CO2/95% air at 37 °C. All cells maintained their broad polygonal morphology during propagation in vitro and were used between passages 4 and 6. The normal LC cells and POAG LC cells were cultured until approximately 90% confluent on 100 mm (p100) plates. The cells were then serum starved for 24 h before being harvested for extraction of total cellular RNA.

### Oligonucleotide microarray analysis

RNA isolation, cDNA synthesis, in vitro transcription and microarray analysis were performed as described previously [16]. Briefly, total RNA was isolated from all LC cells using the RNeasy minicolumn protocol (Qiagen, Valencia, CA). cDNA was synthesized from total RNA using SuperScript Choice Kit (Invitrogen, Paisley, UK). Biotin labeled cRNA was prepared from template cDNA followed by fragmentation and hybridization to Affymetrix HG-U133A arrays as per the Affymetrix protocol (Affymetrix, Santa Clara, CA). Arrays were then washed and fluorescently labeled before scanning with a confocal laser (Affymetrix, CA). Two separate microarray analyses (n=2) using separate human donors were performed for this study. The microarray experimental study design is illustrated in Figure 1. For microarray analysis 1 (Figure 1A), RNA from three separate normal and three separate POAG donors were pooled to one 'normal' and one 'POAG' microarray respectively. For microarray analysis 2 (Figure 1B), RNA from a fourth normal donor was pooled to a second ‘normal’ micorarray and RNA from a fourth POAG donor was pooled to a second ‘POAG’ microarray. A total of four microarrays were used (two normal and two POAG).

Image files were obtained through Affymetrix MAS 5.0 software. Array normalization and pre-processing was performed using Robust multichip average (RMA) to allow global comparison of all four LC cell microarrays. RMA is a function within R statistical software that analyses directly from the Affymetrix microarray *.cel image file [17]. R v2.2 running on Macintosh v10.4 operating system was used with the installed component packages Affy v1.8.1, Tools v2.2 and Biobase v1.8. RMA consists of several steps to background adjust, quantile normalize, log transform and summarize the gene expression values. Background adjustment compensates for non-specific cRNA/probe binding. The average of the lowest 2% of probe cell values in a region of the microarray is taken as the background value for that region and subtracted from all values in that region. There were 16 such regions on each HG-U133A microarray used. Quantile normalization unifies perfect match cRNA/target probe distributions across the arrays. This minimises the effects of variation in the amounts of RNA used, the rates of the microarray hybridization reactions and the conditions of hybridization within the Affymetrix hybridization oven. The final step, summarization, median polishes the Log Base2 transformed probe signal level data. Output from this analysis was exported in a *.csv file format and filtered using Microsoft Excel. Probe sets with low level expression intensities of less than 6.5 were removed from the microarrays and the remaining highly expressed probe set values in both the normal LC cell and POAG LC cell arrays were subtracted to calculate a signal log ratio (SLR). Genes with SLRs greater than +0.5 or less than −0.5 (±1.5 fold change) were taken to identify the reliably differentially expressed genes between normal and POAG LC cells [18].

### Bioinformatic analysis of microarray data

The filtered group of genes with signal log ratios (SLR) of greater than +0.5 or less than −0.5 were annotated and arranged into biologically relevant categories using NIH EASE software. The NIH Expression Analysis and Systematic Explorer (EASE) can identify themes of gene expression within a data set [19]. More precisely EASE uses a variation of the Fisher exact probability (EASE score) to rank functional gene clusters by statistical over-representation (overexpression) by examining the number of individual genes in specific categories relative to all the genes in the same category available for assay on the HG-U133A microarray. EASE converts Affymetrix probe IDs to LocusLink numbers, ensuring that a single gene represented by more than one identifier (as may occur in GenBank) receives only one vote for each of its ontological categories. The statistically overexpressed gene categories in POAG LC cells versus normal LC cells were separated into three broad components of (1) biologic process (2) molecular function, and (3) cellular component.

### Quantitative real-time PCR validation of microarray data

**Figure 2:**
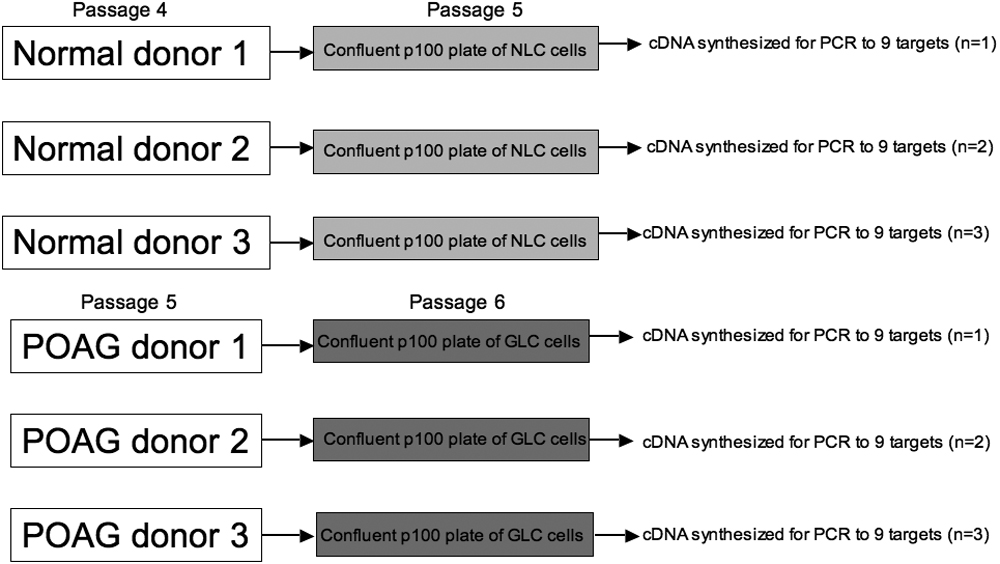
PCR validation assay experimental design. Total RNA (3 μg) was used from three normal and three POAG LC cell lines to synthesize first-strand cDNA. The six cDNA samples (n=3 normal, n=3 POAG) were used for six individual quantitative real-time PCR amplification assays (to 9 targets) with TaqMan^TM^ chemistry.

The normal versus POAG LC cell array data was validated as follows. Total RNA (3 μg) was used from three normal (n=3) and three POAG (n=3) LC cell lines to synthesize first-strand cDNA using random hexamers and SuperScript II reverse transcriptase (Invitrogen, Paisley, UK). The experimental design for these assays is illustrated in Figure 2. The six cDNA samples (n=3 normal, n=3 POAG) were used for six individual quantitative real-time PCR amplification assays for 9 gene targets with TaqMan^TM^ chemistry (Applied Biosystems, Foster City, CA; Figure 2). The fluorogenic probe and sequence specific primers for cartilage linking protein 1 (*CRTL-1*), sulfatase 1 (*SULF1*), bone morphogenetic protein-1 (*BMP-1*), dystrophin (*DMD*), thrombospondin 1 (*THBS1*), periostin (*POSTN*), neuritin 1 (*NRN1*), and prostaglandin D2 synthase (*PTGDS*) with the endogenous control 18S rRNA were designed and optimized as pre-formulated assay reagents (Assay-on-Demand, Applied Biosystems). Duplicate cDNA template samples were amplified and analyzed in the Prism 7900HT sequence detection system (Applied Biosystems). Thermal cycler conditions were 10 min at 95 °C followed by 40 cycles of 30 s at 95 °C to denature the DNA and 30 s at 60 °C to anneal and extend the template. A standard curve of cycle thresholds using serial dilutions of cDNA samples were established and used to calculate the relative abundance of the 9 target genes between normal LC cell and POAG LC cell samples. Values were normalized to the relative amounts of 18S mRNA, which were obtained from a similar standard curve.

### Immunoflourescence histochemistry

Eyes from three human donors were obtained from regional eye banks and placed in 4% neutral buffered formalin within 4 h of death. Two of the donors had a documented history of glaucoma. The third donor had no history of ocular disease (normal control). The posterior segments were embedded in paraffin and 6 μm optic nerve head sections were cut and mounted on glass slides. After paraffin removal, the tissue was quenched for aldehydes by treatment in a 0.05 M glycine (Sigma, Dorset, UK) solution for 15 min. Nonspecific binding sites were then blocked for 15 min with phosphate buffered saline (PBS) solution containing 1% BSA and 1% serum from the species (rabbit) in which the secondary antibody was raised. The slides were washed in PBS before incubation with a mixture of anti-Periostin antibody (diluted 1:25 in PBS; Abcam, Cambridge, MA) at 4 °C overnight. The sections were then washed 3 times in PBS. For the detection of periostin (*POSTN*), the sections were incubated with Alexa Fluor-488 labeled anti-rabbit IgG for 1 h at room temperature (RT) (secondary antibody was obtained from Molecular Probes, Leiden, The Netherlands). The tissue nuclei were then DAPI stained (300 nM in water) for 5–10 min. Images were captured with a Nikon Microphot FXA (Nikon, Inc., Melville, NY) equipped with a SenSys CCD camera (Photometrics, Tucson, AZ). Images were deconvoluted using Scanalytics IPLAB (Scanalytics, Fairfax, VA) and Vaytek Microtome (Vaytek, Fairfield, IA) software.

### Statistical analysis

Data from real time PCR assays were summarized as the mean from three separate experiments (n=3). The paired student *t*-test was used to analyze the statistical significance (*p<0.05) of differences between mean values.

## Results

### Global and ECM transcription differential between POAG and normal LC cells

**Figure 3:**
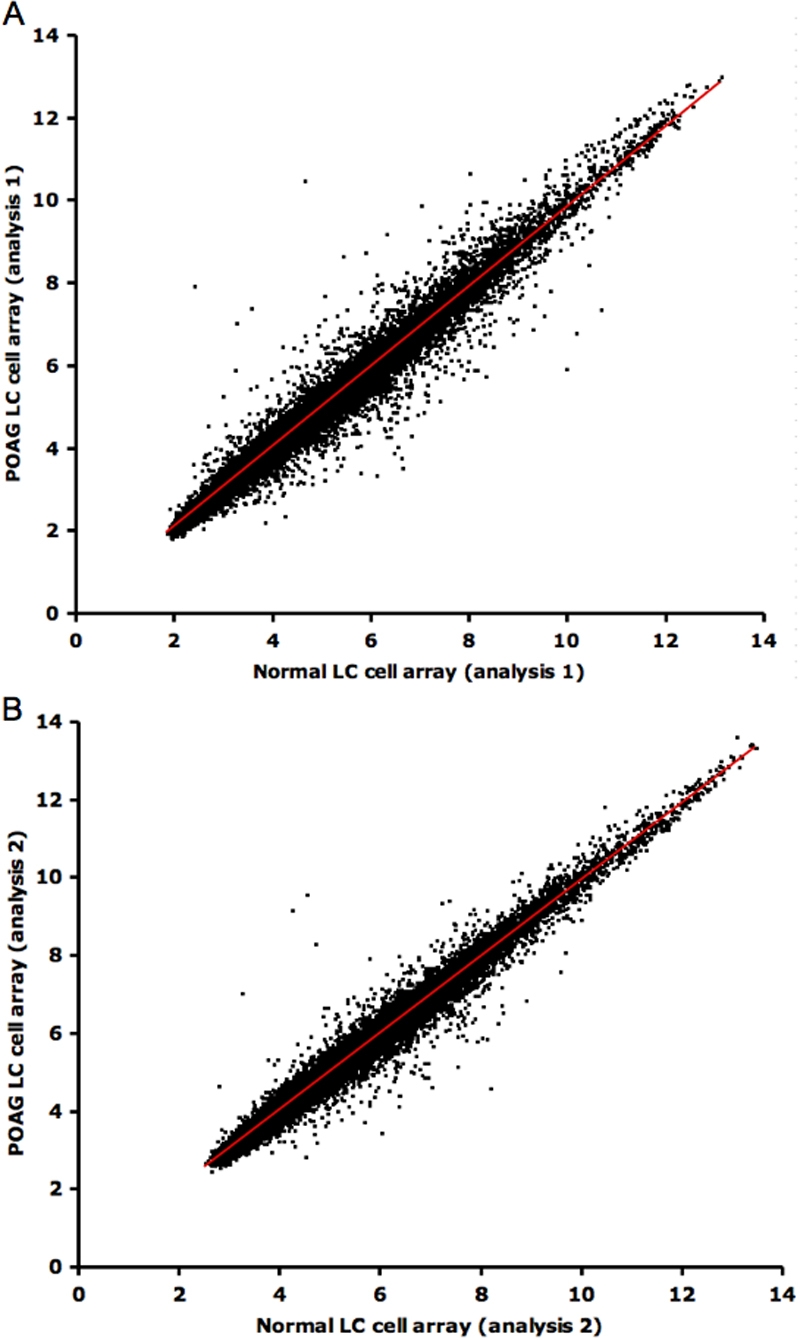
Scatter plot matrices of the two biologically replicated microarray analyses. Panel **A** shows microarray analysis 1. Panel **B** shows microarray analysis 2. In each scatterplot the POAG LC cell versus normal LC cell microarray expression data following normalization with RMA is shown. All 22,283 Log base2 transformed probe (gene) signal intensities (range 0 - 14) for the normal LC arrays are plotted on the x-axes and the corresponding value (range 0 - 14) for the POAG LC arrays on the y-axes. For each probe (point), its position relative to the diagonal identity line (red) directly relates the ratio of expression in POAG versus normal control. Probes that appear above the identity were overexpressed in POAG LC cells (upregulated); probes that appear below the diagonal were overexpressed in the normal LC cells (down regulated). Probes with identical expression levels in both POAG and normal LC cells appear along or on the identity line.

The pair-wise comparison of RMA normalized expression values for all 22,283 mRNA transcripts assayed in the two normal and two POAG microarrays are shown in Figure 3. Microarray analysis 1 is shown in Figure 3A and microarray analysis 2 is shown in Figure 3B. 183 transcripts were upregulated by >+0.5 SLR and 220 were down regulated by <−0.5 SLR in the POAG LC cell transcriptome versus the normal. In each gene comparison, at least one of the probesets had an RMA signal intensity greater than or equal to 6.5. These data are freely available for download at the Gene Expression Omnibus (GEO) database accessions (GSE13534). Appendix 1 summarize the pattern of change within the major gene categories classified on the basis of known function and statistically significant over representation. Among the functional categories most significantly expressed were extracellular matrtix (ECM) (EASE score <0.0001), collagen (EASE score <0.05), and extracellular space (EASE score <0.05).

Table 1 highlights 50 (25 upregulated and 25 down regulated) of the genes whose mRNA levels differed most between POAG and normal LC cells. Among the 25 most upregulated genes in POAG LC cells were periostin (*POSTN*; +3.0 SLR), cartilage linking protein 1 (*CRTL-1*; +1.8 SLR), dystrophin (*DMD*; +2.0 SLR), Rho GDP dissociation inhibitor beta (*ARHGDIB*; +1.1 SLR), sulfatase 1 (*SULF1*; +1.1 SLR), thrombospondin-1 (*THBS1*;0.8 SLR), and bone morphogenetic protein-1 (*BMP-1*; +0.7 SLR). Among the 25 most down regulated genes in POAG were neuritin 1 (*NRN1*; −1.0 SLR) and prostaglandin D2 synthase (*PTGDS*; −1.7 SLR). The ECM gene category, which was significantly overexpressed in POAG versus normal contained 23 genes. These ECM genes are listed in Table 2. The proteins of these ECM genes have previously been documented in POAG in the lamina cribrosa, e.g., collagen type I (*COL1A1*; +0.6 SLR), collagen type V (*COL5A1*; +0.5 SLR) and collagen type XI (*COL11A1*; +0.5 SLR). Notable also were ECM genes or ECM modulating genes not classically associated with POAG changes in the optic nerve head such as tissue inhibitor of matrix metalloproteinase-3 (*TIMP-3*; +1.3 SLR), decorin (*DCN*; −0.9 SLR), versican (*VCAN*; +1.2 SLR), lysyl oxidase (*LOX*; +0.7 SLR), and secreted acid protein cysteine rich (*SPARC*; +1.1 SLR). Validation of the characterization of our LC cells as GFAP-negative was demonstrated by very low signal detection (less than 6.5) for GFAP in both of the normal (mean signal 4.0) and POAG (mean signal 4.0) microarrays (see Table 1).

**Table 1:** Fifty of the most upregulated and down-regulated probe sets (genes) in POAG LC cells versus normal LC cells.

**PROBE ID**	**GENE**	**Mean normal LC array signal (n=2)**	**Mean POAG LC array signal (n=2)**	**SLR (n=2)**
210809_s_at	**periostin**	7.7	10.7	3.0
212951_at	G protein-coupled receptor 116	4.5	6.9	2.5
203881_s_at	**dystrophin (muscular dystrophy, Duchenne and Becker types)**	5.5	7.5	2.0
205523_at	**cartilage linking protein 1**	4.7	6.5	1.8
203441_s_at	cadherin 2, type 1, N-cadherin (neuronal)	6.2	7.8	1.6
201150_s_at	tissue inhibitor of metalloproteinase 3 (TIMP-3)	7.3	8.6	1.3
206046_at	a disintegrin and metalloproteinase domain 23	5.3	6.6	1.3
200974_at	actin, alpha 2, smooth muscle, aorta	10.2	11.5	1.2
212572_at	serine/threonine kinase 38 like	6.7	7.8	1.2
215646_s_at	chondroitin sulfate proteoglycan 2 (versican)	7.3	8.4	1.2
202363_at	sparc/osteonectin, cwcv and kazal-like domains proteoglycan (testican)	8.0	9.1	1.1
202620_s_at	procollagen-lysine, 2-oxoglutarate 5-dioxygenase (lysine hydroxylase) 2	6.9	8.0	1.1
201310_s_at	chromosome 5 open reading frame 13	8.4	9.5	1.1
212354_at	**sulfatase 1**	7.5	8.6	1.1
201288_at	**Rho GDP dissociation inhibitor (GDI) beta**	5.8	6.9	1.1
203440_at	cadherin 2, type 1, N-cadherin (neuronal)	8.0	9.0	1.0
211356_x_at	leptin receptor	5.5	6.5	1.0
221011_s_at	likely ortholog of mouse limb-bud and heart gene	6.8	7.7	0.9
210372_s_at	tumor protein D52-like 1	7.8	8.7	0.9
218717_s_at	myxoid liposarcoma associated protein 4	6.8	7.6	0.8
213869_x_at	Thy-1 cell surface antigen	6.7	7.5	0.8
217853_at	tensin-like SH2 domain-containing 1	6.8	7.6	0.8
201110_s_at	**thrombospondin 1**	8.8	9.6	0.8
201109_s_at	thrombospondin 1	9.8	10.5	0.8
205574_x_at	**bone morphogenetic protein 1**	7.2	7.9	0.7
203540_at	glial fibrillary acidic protein (GFAP)	4.0	4.0	0.0
212187_x_at	**prostaglandin D2 synthase 21 kDa (brain)**	8.9	7.2	−1.7
213880_at	G protein-coupled receptor 49	6.8	5.5	−1.3
209081_s_at	collagen, type XVIII, alpha 1	7.5	6.1	−1.3
204897_at	prostaglandin E receptor 4 (subtype EP4)	6.7	5.4	−1.3
212279_at	hypothetical protein MAC30	7.3	6.0	−1.3
202437_s_at	cytochrome P450, family 1, subfamily B, polypeptide 1	8.9	7.6	−1.3
217997_at	pleckstrin homology-like domain, family A, member 1	6.6	5.3	−1.3
212386_at	transcription factor 4	7.5	6.4	−1.1
209160_at	aldo-keto reductase family 1, member C3	6.8	5.6	−1.3
215034_s_at	transmembrane 4 superfamily member 1	8.8	7.6	−1.3
219064_at	inter-alpha trypsin inhibitor heavy chain precursor 5	7.5	6.3	−1.2
206373_at	Zic family member 1 (odd-paired homolog, Drosophila)	6.9	5.7	−1.2
214022_s_at	interferon induced transmembrane protein 1 (9–27)	7.2	6.0	−1.2
201525_at	apolipoprotein D	6.9	5.7	−1.1
212730_at	desmuslin	7.2	6.1	−1.1
204249_s_at	LIM domain only 2 (rhombotin-like 1)	6.5	5.1	−1.4
209466_x_at	pleiotrophin	8.0	6.9	−1.1
202434_s_at	cytochrome P450, family 1, subfamily B, polypeptide 1	6.7	5.6	−1.0
213891_s_at	transcription factor 4	7.2	6.2	−1.0
218625_at	**neuritin 1**	7.1	6.2	−1.0
208791_at	clusterin	8.0	7.1	−0.9
202075_s_at	phospholipid transfer protein	6.9	6.0	−0.9
209335_at	decorin	7.7	6.9	−0.9
204987_at	inter-alpha (globulin) inhibitor, H2 polypeptide	6.2	5.4	−0.9
212992_at	chromosome 14 open reading frame 78	7.9	7.2	−0.8

Leftmost column: Affymetrix probe IDs; center column: gene names; right most columns: RMA normalized gene signal intensities for normal (n=2) and POAG (n=2) and calculated signal log ratio changes for each gene. Probe IDs were annotated by uploading tab delimited *.txt files of the IDs and signal log ratios to the NIH DAVID software website as described in the Methods section. Genes in bold were validated by real time PCR. Confirmation of the GFAP-negativity of the normal and POAG LC cells is shown by low signal levels for GFAP.

**Table 2:** Extracellular matrix (ECM) probe sets (genes) that were differentially expressed in POAG LC cells versus normal LC cells.

**PROBE ID**	**GENE**	**Mean normal LC array signal (n=2)**	**Mean POAG LC array signal (n=2)**	**SLR (n=2)**
210809_s_at	osteoblast specific factor 2 (periostin)	7.7	10.7	3.0
203881_s_at	dystrophin	5.5	7.5	2.0
205524_s_at	cartilage linking protein 1	4.7	6.5	1.8
201150_s_at	tissue inhibitor of metalloproteinase 3 (TIMP-3)	7.3	8.6	1.3
215646_s_at	chondroitin sulfate proteoglycan 2 (versican)	7.3	8.4	1.2
202363_at	sparc/osteonectin, cwcv and kazal-like domains proteoglycan	8.0	9.1	1.1
211571_s_at	chondroitin sulfate proteoglycan 2 (versican)	7.8	8.8	1.1
201110_s_at	thrombospondin 1	8.8	9.6	0.8
204298_s_at	lysyl oxidase	9.5	10.2	0.7
201842_s_at	EGF-containing fibulin-like extracellular matrix protein 1	9.8	10.4	0.6
201506_at	transforming growth factor, beta-induced, 68 kDa	11.1	11.7	0.6
202311_s_at	collagen, type I, alpha 1	8.7	9.2	0.6
212667_at	secreted protein, acidic, cysteine-rich (osteonectin)	10.1	10.6	0.6
212488_at	collagen, type V, alpha 1	9.6	10.1	0.5
209278_s_at	tissue factor pathway inhibitor 2	6.9	7.4	0.5
204320_at	collagen, type XI, alpha 1	6.8	7.3	0.5
205200_at	tetranectin (plasminogen binding protein)	9.1	7.6	−1.4
209081_s_at	collagen, type XVIII, alpha 1	7.5	6.1	−1.3
203886_s_at	fibulin 2	8.1	7.1	−1.0
211343_s_at	collagen, type XIII, alpha 1	5.7	4.8	−1.0
209335_at	decorin	7.7	6.9	−0.9
202995_s_at	fibulin 1	6.2	5.5	−0.7
212713_at	microfibrillar-associated protein 4	7.2	6.5	−0.7

The individual member genes of the functional family of ECM was annotated in the table above using NIH DAVID software and it lists ECM genes which were up or down regulated by ± 0.5 SLR or greater in POAG LC cells versus normal LC cells.

### Validation of POAG versus normal LC cell microarray analysis using quantitative real time PCR

To validate the normal versus POAG expression patterns obtained from the microarray analyses we investigated the expression of 9 genes using quantitative real time PCR. Given that microarray experiments yield large amounts of data we focused our real time PCR analyses on a sample of targets that were members of the most up or down regulated genes or were members of the ECM category. The validated upregulated targets were: periostin (*POSTN*; 35 fold, *p=0.008), Rho GDP dissociation inhibitor beta (*ARHGDIB*; 4.5 fold, *p=0.02), cartilage linking protein 1 (*CRTL-1*; 240 fold, *p=0.003), dystrophin (*DMD*; 3.2 fold, *p=0.0005), sulfatase 1 (*SULF1*; 8 fold, *p=0.006), thrombospondin-1 (*THBS1*; 1.7 fold, *p=0.02) and bone morphogenetic protein-1 (*BMP-1*; 2.0 fold, *p=0.02). The validated down regulated targets were neuritin 1 (*NRN1*; 60 fold, *p=0.02) and prostaglandin D2 synthase (*PTGDS*; 22 fold, *p=0.02). Table 3 illustrates the results of these analyses with the corresponding microarray fold expression changes determined for each gene. All targets assayed by real time PCR showed a significant change (*p<0.05) which was consistent with the direction of change identified by the microarray analysis.

**Table 3:** Validation of the microarray expression patterns of POAG versus normal LC cells by real time quantitative PCR.

**Gene**	**Microarray analysis**	**Real time PCR**
**Upregulated in POAG**	**Mean fold change (n=2)**	**Mean fold change (n=3)**
periostin	8	35 (*p=0.008)
cartilage linking protein-1	3.5	240 (*p=0.003)
dystrophin	4	3.2 (*p=0.0005)
sulfatase-1	2	8 (*p=0.006)
rho GDP dissociation inhibitor beta	2	4.5 (*p=0.02)
thrombospondin-1	1.7	1.7 (*p=0.02)
BMP-1	1.6	2 (*p=0.02)
		
**Downregulated in POAG**	**Mean fold change (n=2)**	**Mean fold change (n=3)**
neuritin-1	2	60 (*p=002)
prostaglandin D2 synthase	3.2	22 (*p=0.02)

Columns show the gene name, the fold expression change (converted from signal log ratio (SLR) using the formula: fold change=2^(signal log ratio)^) determined by microarray and the corresponding expression change determined by real time PCR. Results for real time PCR are shown in bold and are expressed as the mean of three separate experiments (n=3) with the calculated p value, where *p<0.05 was considered to be statistically significant.

### Immunoflourescence histochemistry

**Figure 4:**
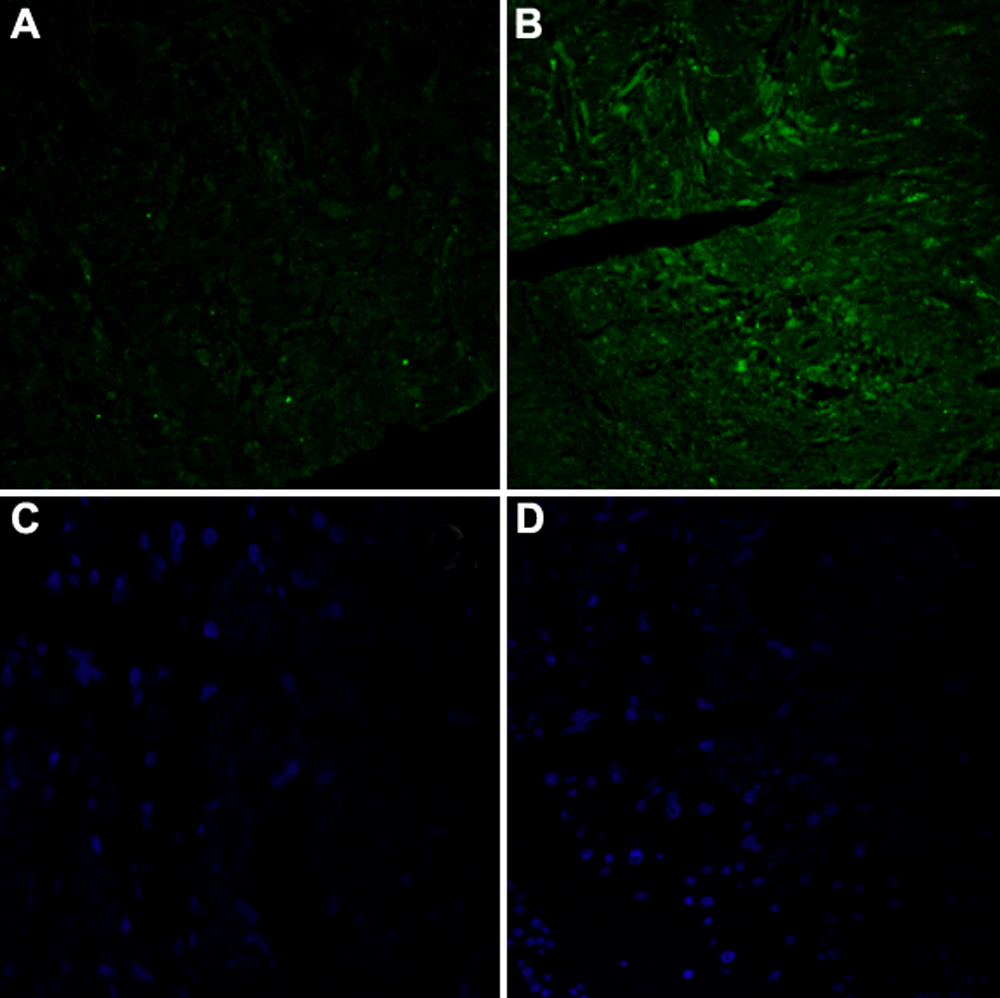
Representative immunofluorescence histochemistry of normal and glaucomatous human optic nerve head lamina cribrosa tissue. Normal (**A**) and glaucomatous (**B**) optic nerve head sections were stained for Periostin (green). Periostin was increased in the lamina cribrosa of glaucomatous sections compared to the normal controls. No immunostaining was seen in the absence of primary antibody (**C** and **D** shows blue DAPI staining of nuclei).

Periostin (*POSTN*; green) immunoreactivity was increased in the lamina cribrosa of glaucomatous optic nerve tissue sections (Figure 4B) compared with the normal control sections (Figure 4A). A representative micrograph is shown in Figure 4. No staining was evident when the primary antibody was omitted (Figure 4C,D).

## Discussion

This study investigated the transcriptional differences between normal and POAG LC cells which we believe contributes substantially to current understanding of ECM remodeling mechanisms in the lamina cribrosa in POAG. We have established for the first time, a baseline transcription differential between the normal and diseased state GFAP-negative LC cell, in terms of both genome-wide and ECM expression. In addition, our objective was to identify whether this transcription differential between POAG and normal LC cells was pro-fibrotic, further supporting the paradigm that LC cells contribute to ECM remodeling in POAG.

In the global gene expression comparison, we identified 403 genes that were reliably differentially expressed in POAG LC cells compared to normal LC cells by greater than ±0.5 signal log ratios with RMA signal intensities of greater than 6.5. EASE analysis further identified that three functional gene categories of ECM, extracellular space and collagen were among the most significantly (*p<0.05) overexpressed categories within this 403-gene data set (Appendix 1). The disease progression of POAG involves not only fibrotic ECM remodeling of the lamina cribrosa, but other pathological phenomena such as reduced optic nerve head blood flow, retinal ganglion cell axon death and transcriptional reactivation of developmentally regulated genes [20-22]. The top 50 most differentially expressed genes in POAG LC cells compared with normals, reflected this multi-mechanistic pathology by containing an interesting combination of ECM, cytoskeletal, angiogenic, neuronal and developmental genes. These included thrombospondin-1 (*THBS1*), prostaglandin D2 synthase (*PTGDS*), sulfatase 1 (*SULF1*), pleiotrophin (*NEGF1*), neuritin 1 (*NRN1*), N-cadherin (*NCAD*), bone morphogenetic protein-1 (*BMP-1*), and zic family member 1 (*ZIC1*; Table 1). In addition, by demonstrating low probe signal levels (below 6.5) for GFAP in both normal and POAG LC cells (Table 1), our microarray output provides further evidence that LC cells are distinguishable from the GFAP-positive optic nerve head astrocyte population of the lamina cribrosa.

Thrombospondin-1 (*THBS1*; upregulated in POAG LC cells) is a major activator of extracellular transforming growth factor beta 1 (*TGFβ1*) in fibrotic renal disease in the rat and is closely involved in angiogenesis in hepatocellular carcinoma [23,24]. Prostaglandin D2 synthase (*PTGDS*; down regulated in POAG LC cells) has an antihypoxic effect in mice [25] which together with thrombospondin-1 upregulation was intriguing in the context of hypoxic insult and vascular hypoperfusion of the optic nerve head in POAG. Sulfatase 1 (*SULF1*) has been shown as an early marker of glial cell precursors [26] and its upregulation in POAG LC cells suggests consistency with the hypothesis that wound formation and healing involves the recapitulation of embryonic gene expression. Pleiotrophin (*NEGF1*) down regulation and neuritin 1 (*NRN1*) upregulation in POAG LC cells was also interesting in terms of axon death in POAG as the primary function of these genes are in neuritogenesis [27,28]. N-cadherin (*NCAD*) which was upregulated in POAG LC cells, is involved in synaptic adhesion in the CNS and is transcriptionally regulated by TGFβ [29,30]. Bone morphogenetic protein-1 (*BMP-1*) and zic family member 1 (*ZIC1*), two other developmental genes, play roles in ECM synthesis and CNS development, respectively [31,32].

The results of EASE analysis clearly stated that ECM was a significant gene family difference between normal and POAG LC cells. A total of 23 ECM genes were differentially expressed by greater than +/−0.5 SLR in POAG versus normal LC cells (Table 1). This provided transcriptional evidence that the LC cell may be a source of fibrotic ECM protein production in the glaucomatous lamina cribrosa, particularly as this collection of genes contained the classic fibrotic marker, collagen type I [33-35]. Others, which included cartilage linking protein 1 (*CRTL-1*), periostin (*POSTN*), and dystrophin (*DMD*), are novel in the context of optic nerve head remodeling in POAG.

Our finding that periostin (*POSTN*) was upregulated at the mRNA level in POAG LC cells and at the protein level in optic nerve head tissue was interesting in that this gene is regulated by hypoxia responsive growth factors (e.g., FGF-1) and is thought to be one of the main genes involved in ECM tissue remodeling following mechanical stress in rat pulmonary arterial smooth muscle cells and human cardiac myocytes [36-38]. This demonstrates consistency with the established mechanical and vascular theories of optic nerve damage in POAG. Furthermore, our data are consistent with that of a previous animal model study which demonstrated a linear increase in optic nerve head tissue periostin (*POSTN*) mRNA expression in response to elevated IOP [39].

Cartilage linking protein 1 (*CRTL-1*) which was also upregulated in POAG LC cells, stabilizes the interaction between hyaluronan and the ECM [40]. Hyaluronans provide connective tissues with mechanical resilience and are important components of the human lamina cribrosa [5]. Upregulation of *CRTL-1* may represent an attempt to mechanically re-inforce the lamina cribrosa against rising IOP or inhibit falling hyaluronan levels which have also been shown in POAG optic nerve heads [41]. Dystrophin (*DMD*) upregulation in POAG LC cells may also bear relevance to transduction of mechanical stimuli in raised IOP, as the protein of this gene forms links between the ECM and the cytoskeleton [42]. Tissue inhibitor of matrix metalloproteinase-3 (*TIMP-3*) is a member of the tissue inhibitor of matrix metalloproteinase family (TIMPs). The protein of this gene specifically inhibits several ECM degrading enzymes including matrix metalloproteinase-2 (*MMP-2*) [43]. The substrates for MMP-2 include elastin and collagen type IV [44]. Upregulated expression of TIMP-3 in our POAG LC cells is, therefore, consistent with the overall increase in collagen type IV (*COL4A1*) and elastin that is observed in the lamina cribrosa in POAG. Decorin down regulation in our POAG LC cells may also be of significance to loss of lamina cribrosa architecture in POAG. It co-localizes with collagen type I (*COL1A1*) in the ECM modulating collagen fiber spacing and assembly [45].

Transforming growth factor beta induced (TGFβI), was another upregulated ECM gene in our POAG LC cells that plays a role in collagen metabolism. We have previously shown that TGFβI is a transforming growth factor beta-1 (TGFβ-1) inducible gene in LC cells [13]. The protein of this gene binds and promotes aggregation of collagen type VI (*COL6A1*) and mediates cell-collagen interactions in the ECM [46]. Since type VI collagen is a major component of the lamina cribrosa, TGFβI overexpression in POAG LC cells may explain the accumulation of type VI collagen in the glaucomatous lamina cribrosa. Other noteworthy upregulated ECM genes in POAG LC cells in our study included versican (*VCAN*) whose protein is synonymous with the fibroblastic phenotype [47,48]. In addition, the ECM-related gene lysyl oxidase (*LOX*), which initiates the cross linking of collagen and elastin was also upregulated in our system. Interestingly, another member of this gene family lysyl oxidase-like 1 (*LOXL1*) has been found to contain two single nucleotide polymorphisms (SNPs) in patients who develop pseudoexfoliation glaucoma [49].

In summary, this work has identified a baseline transcriptional differential between GFAP-negative LC cells from normal and POAG human donors in-vitro. This transcriptional differential is strikingly defined by pro-fibrotic/ECM genes, which we believe are pathologically characteristic of POAG LC cells in-vivo. The data, therefore, underlines the strong potential role for LC cells in glaucomatous optic nerve head ECM remodeling. POAG LC cells may also possess an ab-initio inevitability to express fibrotic/ECM genes following exposure to other pathological stimuli in POAG such as mechanical strain or hypoxic stress. In conclusion, this analysis provides a framework upon which to base further pathway-specific and LC cell-targeted investigations of the discrete mechanisms that dictate the evolution of optic nerve head ECM remodeling in POAG.

## Appendix 1. Ontology of up and down regulated genes in POAG LC cells versus normal LC cells.

Table **A** lists the ontological categories (biologic process, cellular component, molecular function) generated from the upregulated genes in POAG versus normal LC cells. Table **B** lists the ontological categories generated from the down regulated genes in POAG versus normal LC cells. The significantly overexpressed functional groups are shown. EASE scores (Pvalue) are indicated next to each functional group. EASE scores less than 0.05 were considered statistically significant. To access the data, click or select the words “Appendix 1.” This will initiate the download of a compressed (pdf) archive that contains the file.

## References

[1] Quigley HA (1996). Number of people with glaucoma worldwide.. Br J Ophthalmol.

[2] Flammer J, Orgul S, Costa VP, Orzalesi N, Krieglstein GK, Serra LM, Renard JP, Stefánsson E (2002). The impact of ocular blood flow in glaucoma.. Prog Retin Eye Res.

[3] Morrison JC, Johnson EC, Cepurna W, Jia L (2005). Understanding mechanisms of pressure-induced optic nerve damage.. Prog Retin Eye Res.

[4] Hernandez MR, Ye H (1993). Glaucoma: changes in extracellular matrix in the optic nerve head.. Ann Med.

[5] Hernandez MR, Pena JD (1997). The optic nerve head in glaucomatous optic neuropathy.. Arch Ophthalmol.

[6] Burgoyne CF, Downs JC, Bellezza AJ, Suh JK, Hart RT (2005). The optic nerve head as a biomechanical structure: a new paradigm for understanding the role of IOP-related stress and strain in the pathophysiology of glaucomatous optic nerve head damage.. Prog Retin Eye Res.

[7] Edwards ME, Good TA (2001). Use of a mathematical model to estimate stress and strain during elevated pressure induced lamina cribrosa deformation.. Curr Eye Res.

[8] Quigley HA, Addicks EM (1980). Chronic experimental glaucoma in primates. II. Effect of extended intraocular pressure elevation on optic nerve head and axonal transport.. Invest Ophthalmol Vis Sci.

[9] Hernandez MR (2000). The optic nerve head in glaucoma: role of astrocytes in tissue remodeling.. Prog Retin Eye Res.

[10] Hernandez MR, Igoe F, Neufeld AH (1988). Cell culture of the human lamina cribrosa.. Invest Ophthalmol Vis Sci.

[11] Naugle JE, Olson ER, Zhang X, Mase SE, Pilati CF, Maron MB, Folkesson HG, Horne WI, Doane KJ, Meszaros JG (2006). Type VI collagen induces cardiac myofibroblast differentiation: implications for postinfarction remodeling.. Am J Physiol Heart Circ Physiol.

[12] Lambert W, Agarwal R, Howe W, Clark AF, Wordinger RJ (2001). Neurotrophin and neurotrophin receptor expression by cells of the human lamina cribrosa.. Invest Ophthalmol Vis Sci.

[13] Kirwan RP, Leonard MO, Murphy M, Clark AF, O'Brien CJ (2005). Transforming growth factor-beta-regulated gene transcription and protein expression in human GFAP-negative lamina cribrosa cells.. Glia.

[14] Yang J, Yang P, Tezel G, Patil RV, Hernandez MR, Wax MB (2001). Induction of HLA-DR expression in human lamina cribrosa astrocytes by cytokines and simulated ischemia.. Invest Ophthalmol Vis Sci.

[15] Yang JL, Neufeld AH, Zorn MB, Hernandez MR (1993). Collagen type I mRNA levels in cultured human lamina cribrosa cells: effects of elevated hydrostatic pressure.. Exp Eye Res.

[16] Leonard MO, Cottell DC, Godson C, Brady HR, Taylor CT (2003). The role of HIF-1 alpha in transcriptional regulation of the proximal tubular epithelial cell response to hypoxia.. J Biol Chem.

[17] Bolstad BM, Irizarry RA, Astrand M, Speed TP (2003). A comparison of normalization methods for high density oligonucleotide array data based on variance and bias.. Bioinformatics.

[18] Warrenfeltz S, Pavlik S, Datta S, Kraemer ET, Benigno B, McDonald JF (2004). Gene expression profiling of epithelial ovarian tumours correlated with malignant potential.. Mol Cancer.

[19] Hosack DA, Dennis GJ, Sherman BT, Lane HC, Lempicki RA (2003). Identifying biological themes within lists of genes with EASE.. Genome Biol.

[20] Kerrigan-Baumrind LA, Quigley HA, Pease ME, Kerrigan DF, Mitchell RS (2000). Number of ganglion cells in glaucoma eyes compared with threshold visual field tests in the same persons.. Invest Ophthalmol Vis Sci.

[21] Ricard CS, Pena JD, Hernandez MR (1999). Differential expression of neural cell adhesion molecule isoforms in normal and glaucomatous human optic nerve heads.. Brain Res Mol Brain Res.

[22] Wordinger RJ, Agarwal R, Talati M, Fuller J, Lambert W, Clark AF (2002). Expression of bone morphogenetic proteins (BMP), BMP receptors, and BMP associated proteins in human trabecular meshwork and optic nerve head cells and tissues.. Mol Vis.

[23] Daniel C, Wiede J, Krutzsch HC, Ribeiro SM, Roberts DD, Murphy-Ullrich JE, Hugo C (2004). Thrombospondin-1 is a major activator of TGF-beta in fibrotic renal disease in the rat in vivo.. Kidney Int.

[24] Poon RT, Chung KK, Cheung ST, Lau CP, Tong SW, Leung KL, Yu WC, Tuszynski GP, Fan ST (2004). Clinical significance of thrombospondin 1 expression in hepatocellular carcinoma.. Clin Cancer Res.

[25] Yang X, Sheares KK, Davie N, Upton PD, Taylor GW, Horsley J, Wharton J, Morrell NW (2002). Hypoxic induction of cox-2 regulates proliferation of human pulmonary artery smooth muscle cells.. Am J Respir Cell Mol Biol.

[26] Braquart-Varnier C, Danesin C, Clouscard-Martinato C, Agius E, Escalas N, Benazeraf B, Ai X, Emerson C, Cochard P, Soula C (2004). A subtractive approach to characterize genes with regionalized expression in the gliogenic ventral neuroepithelium: identification of chick sulfatase 1 as a new oligodendrocyte lineage gene.. Mol Cell Neurosci.

[27] Di Giovanni S, De Biase A, Yakovlev A, Yakovlev A, Finn T, Beers J, Hoffman EP, Faden AI (2005). In vivo and in vitro characterization of novel neuronal plasticity factors identified following spinal cord injury.. J Biol Chem.

[28] Hida H, Jung CG, Wu CZ, Kim HJ, Kodama Y, Masuda T, Nishino H (2003). Pleiotrophin exhibits a trophic effect on survival of dopaminergic neurons in vitro.. Eur J Neurosci.

[29] Tanriover G, Kayisli UA, Demir R, Pestereli E, Karaveli S, Demir N (2004). Distribution of N-cadherin in human cerebral cortex during prenatal development.. Histochem Cell Biol.

[30] Tuli R, Tuli S, Nandi S, Huang X, Manner PA, Hozack WJ, Danielson KG, Hall DJ, Tuan RS (2003). Transforming growth factor-beta-mediated chondrogenesis of human mesenchymal progenitor cells involves N-cadherin and mitogen-activated protein kinase and Wnt signaling cross-talk.. J Biol Chem.

[31] Ge G, Seo NS, Liang X, Hopkins DR, Hook M, Greenspan DS (2004). Bone morphogenetic protein-1/tolloid-related metalloproteinases process osteoglycin and enhance its ability to regulate collagen fibrillogenesis.. J Biol Chem.

[32] Yokota N, Aruga J, Takai S, Yamada K, Hamazaki M, Iwase T, Sugimura H, Mikoshiba K (1996). Predominant expression of human zic in cerebellar granule cell lineage and medulloblastoma.. Cancer Res.

[33] di Mola FF, Friess H, Martignoni ME, Di Sebastiano P, Zimmermann A, Innocenti P, Graber H, Gold LI, Korc M, Büchler MW (1999). Connective tissue growth factor is a regulator for fibrosis in human chronic pancreatitis.. Ann Surg.

[34] Stallmach A, Schuppan D, Riese HH, Matthes H, Riecken EO (1992). Increased collagen type III synthesis by fibroblasts isolated from strictures of patients with Crohn's disease.. Gastroenterology.

[35] Hernandez MR, Ye H, Roy S (1994). Collagen type IV gene expression in human optic nerve heads with primary open angle glaucoma.. Exp Eye Res.

[36] Li P, Oparil S, Feng W, Chen YF (2004). Hypoxia-responsive growth factors upregulate periostin and osteopontin expression via distinct signaling pathways in rat pulmonary arterial smooth muscle cells.. J Appl Physiol.

[37] Litvin J, Blagg A, Mu A, Matiwala S, Montgomery M, Berretta R, Houser S, Margulies K (2006). Periostin and periostin-like factor in the human heart: possible therapeutic targets.. Cardiovasc Pathol.

[38] Rios H, Koushik SV, Wang H, Wang J, Zhou HM, Lindsley A, Rogers R, Chen Z, Maeda M, Kruzynska-Frejtag A, Feng JQ, Conway SJ (2005). periostin null mice exhibit dwarfism, incisor enamel defects, and an early-onset periodontal disease-like phenotype.. Mol Cell Biol.

[39] Johnson EC, Jia L, Cepura WO, Doser TA, Morrison JC (2007). Global changes in optic nerve head gene expression after exposure to elevated intraocular pressure in a rat glaucoma model.. Invest Ophthalmol Vis Sci.

[40] Spicer AP, Joo A, Bowling RAJ (2003). A hyaluronan binding link protein gene family whose members are physically linked adjacent to chondroitin sulfate proteoglycan core protein genes: the missing links.. J Biol Chem.

[41] Gong H, Ye W, Freddo TF, Hernandez MR (1997). Hyaluronic acid in the normal and glaucomatous optic nerve.. Exp Eye Res.

[42] Ilsley JL, Sudol M, Winder SJ (2001). The interaction of dystrophin with beta-dystroglycan is regulated by tyrosine phosphorylation.. Cell Signal.

[43] Butler GS, Apte SS, Willenbrock F, Murphy G (1999). Human tissue inhibitor of metalloproteinases 3 interacts with both the N- and C-terminal domains of gelatinases A and B. Regulation by polyanions.. J Biol Chem.

[44] Okada Y, Morodomi T, Enghild JJ, Suzuki K, Yasui A, Nakanishi I, Salvesen G, Nagase H (1990). Matrix metalloproteinase 2 from human rheumatoid synovial fibroblasts. Purification and activation of the precursor and enzymic properties.. Eur J Biochem.

[45] Vesentini S, Redaelli A, Montevecchi FM (2005). Estimation of the binding force of the collagen molecule-decorin core protein complex in collagen fibril.. J Biomech.

[46] Reinboth B, Thomas J, Hanssen E, Gibson MA (2006). Beta ig-h3 interacts directly with biglycan and decorin, promotes collagen VI aggregation, and participates in ternary complexing with these macromolecules.. J Biol Chem.

[47] Hesselstrand R, Westergren-Thorsson G, Scheja A, Wildt M, Akesson A (2002). The association between changes in skin echogenicity and the fibroblast production of biglycan and versican in systemic sclerosis.. Clin Exp Rheumatol.

[48] Lallier TE, Spencer A, Fowler MM (2005). Transcript profiling of periodontal fibroblasts and osteoblasts.. J Periodontol.

[49] Thorleifsson G, Magnusson KP, Sulem P, Walters GB, Gudbjartsson DF, Stefansson H, Jonsson T, Jonasdottir A, Jonasdottir A, Stefansdottir G, Masson G, Hardarson GA, Petursson H, Arnarsson A, Motallebipour M, Wallerman O, Wadelius C, Gulcher JR, Thorsteinsdottir U, Kong A, Jonasson F, Stefansson K (2007). Common sequence variants in the LOXL1 gene confer susceptibility to exfoliation glaucoma.. Science.

